# Supplementary material for: Revealing Reassortment in Influenza A Viruses with TreeSort
Source: Mol Biol Evol. 2025 Aug 8;42(8):msaf133. doi: 10.1093/molbev/msaf133 (PMC12342482; doi:10.1093/molbev/msaf133)
Supplement: msaf133_Supplementary_Data [file msaf133_supplementary_data.pdf]

Supplemental Material for Revealing reassortment in influenza A viruses with TreeSort

Alexey Markin[1,*], Catherine A. Macken[2], Amy L. Baker[1], Tavis K. Anderson[1]

[1] *Virus and Prion Research Unit, National Animal Disease Center, USDA-ARS, USA*

[2] *Bioinformatics Institute, University of Auckland, New Zealand*

**Email: alexey.markin@usda.gov*

Supplemental Figures and Tables for "Revealing reassortment in influenza A viruses with TreeSort".

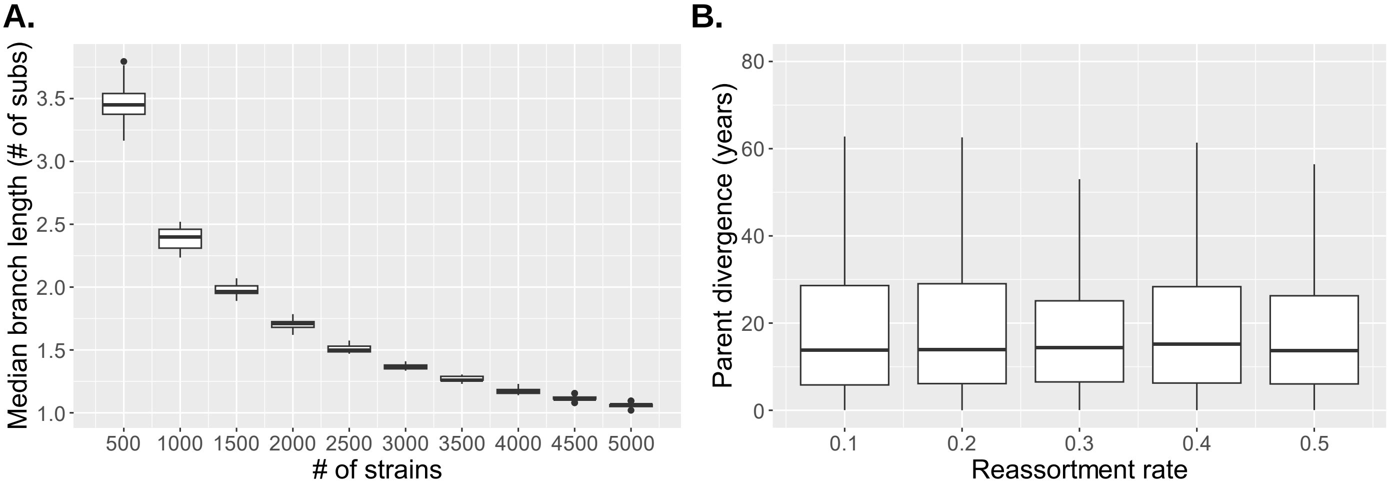

Figure S1: Observed parameters of the simulated data. **A:** The sampling density (median branch length) of the datasets in the simulation study. The median branch length, measured in the number of substitutions per branch, provides a standardized metric comparable across different datasets, and it decreases as the sampling density increases. In simulations, we show that TreeSort is very accurate on datasets with the median branch length below 2. **B:** The distribution of divergence times between pairs of strains involved in reassortment events on the simulated data. The distribution did not significantly differ across the reassortment rates and covered a wide range of divergence times.

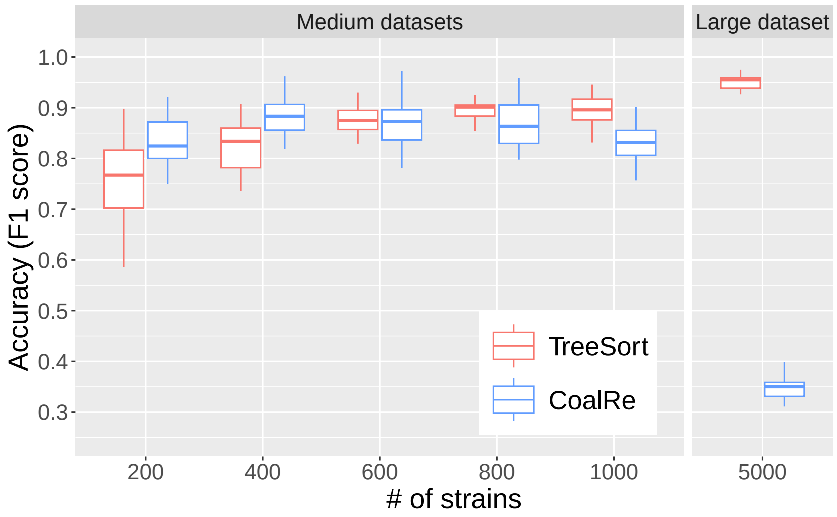

Figure S2: The accuracy comparison between TreeSort and CoalRe by Muller et al. on simulated data. The accuracy is measured in terms of the F1 score that combines the precision and recall metrics [2].

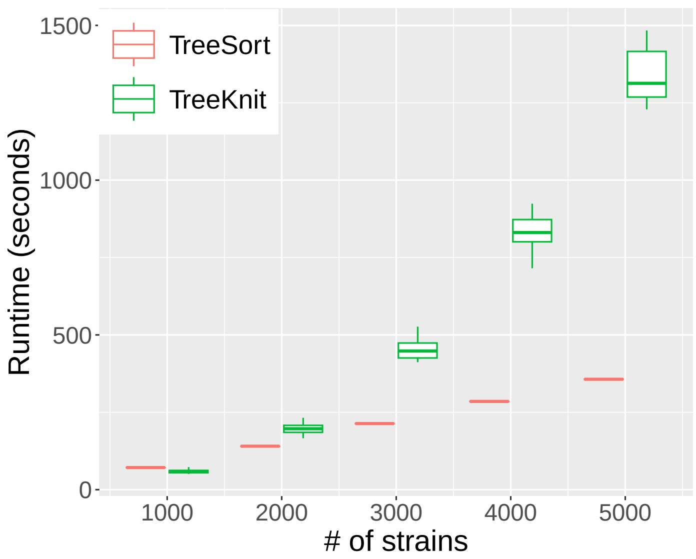

Figure S3: The runtime comparison between TreeSort and TreeKnit on simulated datasets with the number of strains between 1000 and 5000. TreeSort showed a linear increase in runtime with the increase in the dataset size and was significantly faster than TreeKnit on larger datasets.

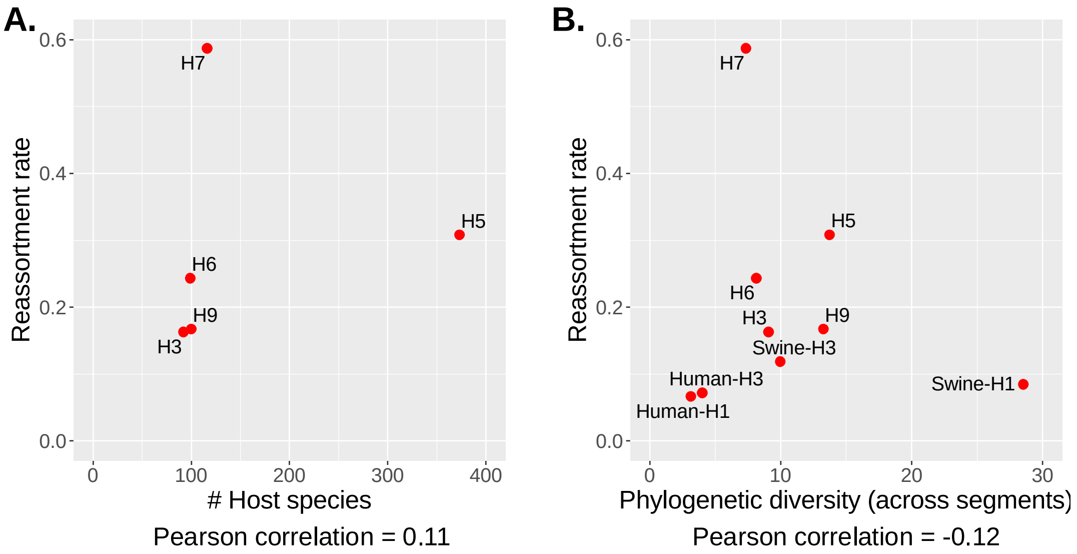

Figure S4: The relationship between reassortment rate and genetic and host diversity of IAV subtypes. **A:** The relationship between the number of distinct host species with IAV detections and the reassortment rate across avian IAV subtypes. **B:** The relationship between the phylogenetic diversity coefficient average across all 8 segments and the reassortment rate. Phylogenetic diversity is a standard measure of genetic diversity computed on a phylogenetic tree [1].

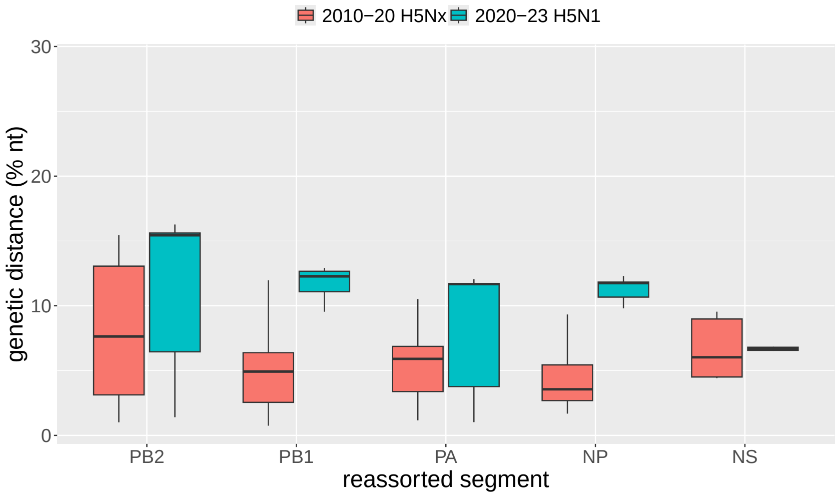

Figure S5: The distance between the genes swapped by reassortment events in two different periods of H5Nx 2.3.4.4b evolution. For example, a genetic distance of 10% for a PB2 gene implies that a single reassortment event occurred where the novel PB2 gene was 10% different from the original PB2. Overall, the H5N1 2.3.4.4b virus in the 2020-2023 period underwent more significant reassortment events than H5Nx 2.3.4.4b in the 2010-2020 period. We omitted NA and MP segments from the comparison due to the low number of reassortment events with these segments in the 2020-2023 period.

Table S1: Substitution rates per site per year for H5Nx clade 2.3.4.4b viruses in two different periods (2010-2020 and 2020-2023) estimated by TreeTime v.0.11.3.

Segment	2010-2020 subst. rate	2020-2023 subst. rate
PB2	0.0020	0.0035
PB1	0.0024	0.0039
PA	0.0023	0.0055
HA	0.0045	0.0055
NP	0.0018	0.0040
NA	0.0027	0.0063
MP	0.0027	0.0061
NS	0.0050	0.0067

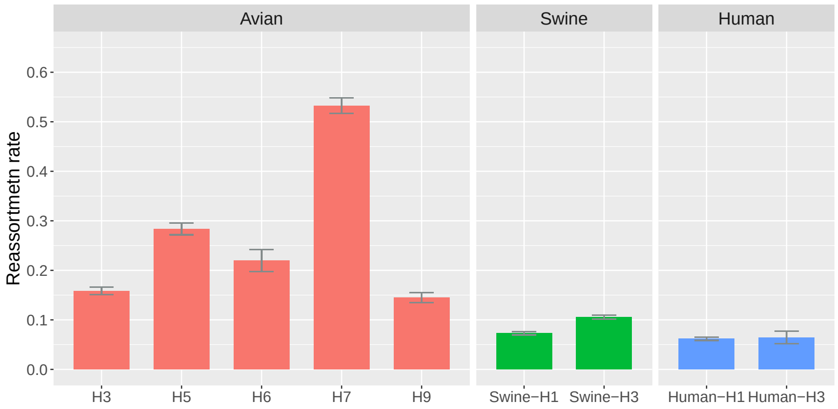

Figure S6: Reassortment rate estimates via the $\hat{\rho}_1$ estimator for major avian, swine, and human subtypes. The reassortment patterns are similar to those shown in Fig. 3 with the $\hat{\rho}_2$ estimator.

References

[1] D. P. Faith. Conservation evaluation and phylogenetic diversity. *Biological conservation*, 61(1):1–10, 1992.

[2] D. M. Powers. Evaluation: from precision, recall and f-measure to roc, informedness, markedness and correlation. *arXiv preprint arXiv:2010.16061*, 2020.
